# Intratracheal myriocin enhances allergen‐induced Th2 inflammation and airway hyper‐responsiveness

**DOI:** 10.1002/iid3.110

**Published:** 2016-06-02

**Authors:** Ramakrishna Edukulla, Kira Lee Rehn, Bo Liu, Jaclyn W. McAlees, Gurjit K. Hershey, Yui Hsi Wang, Ian Lewkowich, Andrew W. Lindsley

**Affiliations:** ^1^ Division of Allergy and ImmunologyCincinnati Children's Hospital Medical CenterCincinnatiOhioUSA; ^2^ Division of ImmunobiologyCincinnati Children's Hospital Medical CenterCincinnatiOhioUSA; ^3^ Division of Asthma ResearchCincinnati Children's Hospital Medical CenterCincinnatiOhioUSA; ^4^ Department of PediatricsUniversity of CincinnatiCincinnatiOhioUSA

**Keywords:** Allergic airway inflammation, ceramide, myriocin

## Abstract

**Introduction:**

Ceramide is the central substrate of sphingolipid metabolism and plays a key role in cellular signal transduction pathways, regulating apoptosis, differentiation, and chemotaxis. Alterations in airway ceramide levels are observed in multiple pulmonary diseases and recent human genetic association studies have linked dysregulation of sphingolipid regulatory genes with asthma pathogenesis.

**Methods:**

Utilizing myriocin, a potent inhibitor of sphingolipid synthesis, we evaluated the immune regulatory role of *de novo* ceramide generation *in vitro* and *in vivo*. Intratracheal myriocin was administered alone or during house dust mite sensitization (HDM) of BALB/C mice and airway hyper‐responsiveness (AHR) was evaluated by invasive plethysmography followed by bronchial lavage (BAL) cytology and cytokine quantification.

**Results:**

Myriocin inhibits and HDM exposure activates *de novo* ceramide synthesis in bone marrow‐derived dendritic cells. Mice receiving intratracheal myriocin developed a mild airway neutrophilic infiltrate without inducing a significant increase in AHR. CXCL1 was elevated in the BAL fluid of myriocin‐treated mice while the neutrophilic chemotactic factors anaphylatoxin C5a, leukotriene B4, and IL‐17 were unaffected. HDM treatment combined with myriocin led to a dramatic enhancement of AHR (63% increase over HDM alone, *p* < 0.001) and increased granulocyte pulmonary infiltrates versus HDM or myriocin alone. Elevated Th2 T cell counts and Th2 cytokines/chemokines (IL5, IL13, CCL17) were observed in mice treated with combined HDM/myriocin compared to HDM alone. Myriocin‐treated pulmonary CD11c+ cells stimulated with HDM secreted significantly more CXCL1 than cells stimulated with HDM alone while HDM stimulated airway epithelial cells showed no change in CXCL1 secretion following myriocin treatment.

**Conclusions:**

Intratracheal myriocin, likely acting via ceramide synthesis inhibition, enhances allergen‐induced airway inflammation, granulocyte and Th2 lymphocyte recruitment, and allergen‐induced AHR. Sphingolipid pathways may represent novel targets for possible future anti‐inflammatory asthma medications.

## Introduction

Approximately 70% of all asthma patients suffer from the allergic subtype of the disease 1. Many individuals initially develop wheezing, the central symptom of asthma, during early childhood, but only a fraction of these patients will develop the chronic airway hyper‐responsiveness (AHR) that defines persistent asthma 2. In children, sensitization to perennial indoor aeroallergens generally precedes sensitization to common seasonal outdoor allergens, and these early allergen exposures may play an important role in pediatric asthma pathogenesis 3. House dust mite (HDM) sensitization is the most commonly detected environmental allergy among young children, and this sensitization increases the lifetime risk of developing asthma 3, 4.

The pathogenesis of pediatric asthma is secondary to both genetic predisposition and environmental/infectious exposures 5. Multiple, well‐powered genome‐wide association studies (GWAS) have linked the human 17q21 chromosomal region to pediatric‐onset asthma 6, 7, 8. The most significant, asthma‐associated single‐nucleotide polymorphisms (SNPs) in the 17q21 locus are associated with over‐expression of a minimally studied gene named ORMDL sphingolipid biosynthesis regulator 3 (*ORMDL3*), which encodes ORM1‐like protein 3 (ORML3) 9. Over‐expression of *ORMDL3* in a transgenic mouse model led to increases in AHR, airway inflammation, goblet cell metaplasia, and basal immunoglobulin E (IgE) levels 10. However, the mechanisms of how ORML3 over‐expression enhances asthma pathogenesis remain unclear. ORML3 is a widely expressed endoplasmic reticulum‐resident transmembrane protein that inhibits the activity of serine palmitoyltransferase (SPT), the rate‐limiting enzyme in *de novo* ceramide synthesis 11, 12. Ceramide, an acyclic aliphatic waxy lipid, is the prototypic sphingolipid, a critical class of signaling molecule with complex roles in cellular signal transduction 13.

**Figure 1:**
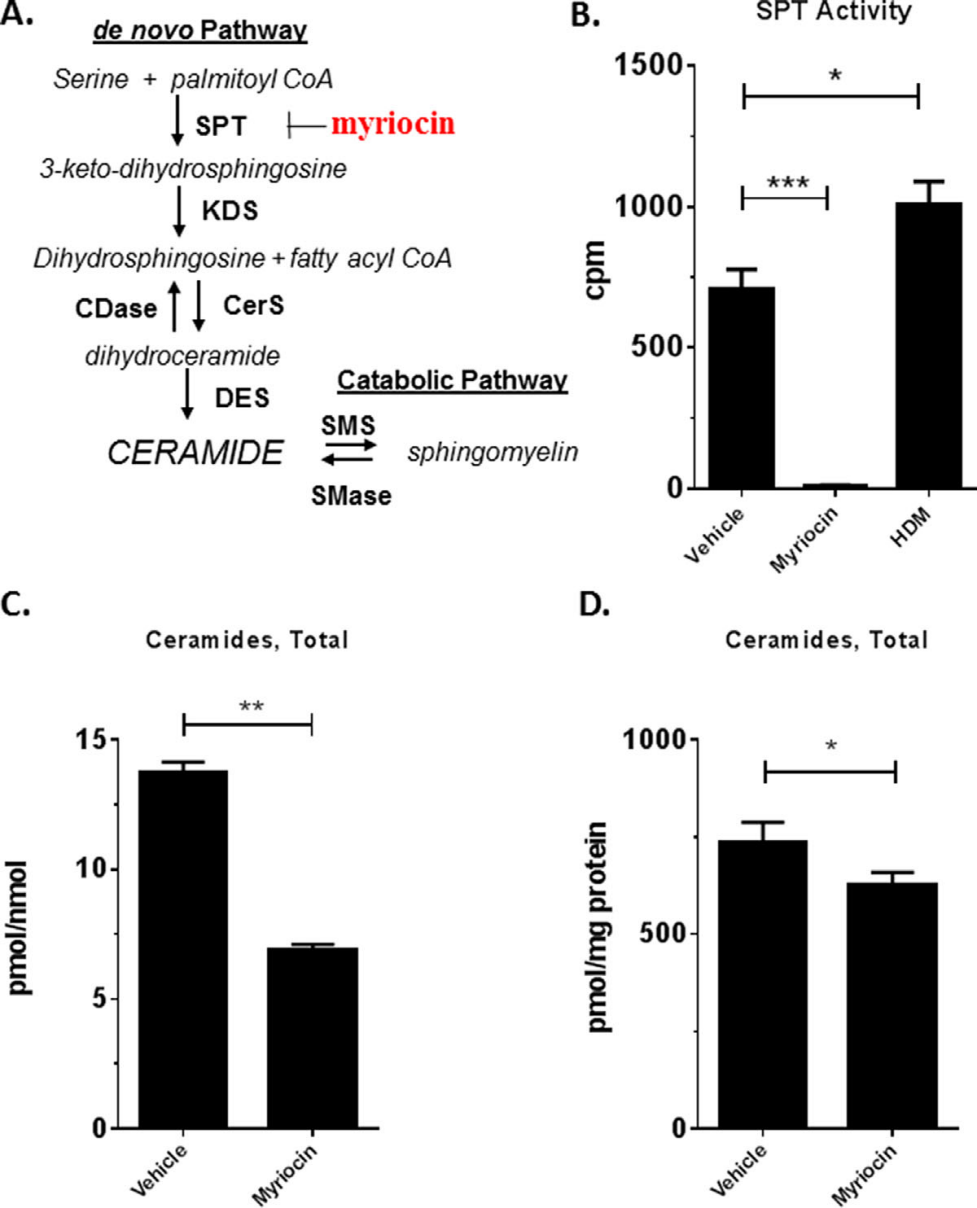
Ceramide metabolism. (A) Ceramide *de novo* and catabolic synthesis pathways. (B) SPT activity in BMDCs; myriocin (20 μM, 8 h) or HDM (30 μg/mL, 8 h) treatment (performed in triplicate, two independent experiments). (C) Steady‐state total ceramide in NHBE cells normalized to phospholipid (Pi); ±vehicle or myriocin, 24 h (performed in triplicate, two independent experiments). (D) Steady‐state total ceramide in whole lung, normalized to total protein, 24 h after vehicle or myriocin (1.2 μg/dose, *n* = 16/group). Data presented as mean ±SEM. (*p < 0.05, ***p < 0.001). SPT, serine palmitoyltransferase; KDS, 3‐ketodihydrosphingosine reductase; CDase, ceramidase; CerS, ceramide synthases; DES, dihydroceramide desaturase; SMS, sphingomyelin synthase; SMase, sphingomyelinase; BMDC, bone marrow‐derived dendritic; cells; cpm, counts per minute; NHBE, normal human bronchial epithelial cells.

Sphingolipid signaling regulates key mechanisms of cell proliferation, apoptosis, differentiation, and chemotaxis 14. Given their role in signaling, intracellular concentrations of simple sphingolipids, such a ceramide, sphingosine, ceramide‐1‐phosphate, and sphingosine‐1‐phosphate (S1P), are low at baseline, whereas complex sphingolipids, such as sphingomyelin (SM) and glycosphingolipids, are present at 10–100 times higher levels and represent the majority of cellular sphingolipids 15, 16. Cellular ceramide levels are dynamic and rapidly increase after exposure to a wide‐range of stimuli including inflammatory mediators (lipopolysaccharide [LPS], tumor necrosis factor alpha [TNF‐α]) and cellular stress (oxidative stress, serum deprivation) 17, 18, 19. Increases of intracellular ceramide are mediated by two major pathways—*de novo* synthesis catalyzed by the SPT enzyme and catabolism of SM via various sphingomylinases (Fig. 1A) 20. Increased airway ceramide levels have been implicated in the pathogenesis of pulmonary diseases such as emphysema and cystic fibrosis, but little is known about the specific roles of ceramide signaling in asthma pathogenesis 21, 22. A recent study in mice showed that both pharmacologic inhibition (myriocin) and genetic impairment (*Sptlc2* haploinsufficiency) of *de novo* ceramide production increased AHR in response to methacholine challenge 23. Myriocin (ISP‐1; thermozymocidin) is a potent inhibitor of *de novo* ceramide synthesis which physically binds to and inhibits the SPT holoenzyme 24.

To investigate myriocin's anti‐SPT effects on *in vivo* allergen sensitization, we utilized a murine model of allergic asthma where intratracheal myriocin was administered during aeroallergen sensitization. We noted significant changes in granulocyte and T cell chemotaxis, as well as enhanced airway resistance, after antigen challenge in HDM‐sensitized mice co‐treated with myriocin. We also show *in vitro* that exogenous ceramide supplementation attenuates pro‐inflammatory signaling while myriocin treatment enhances cytokine production in pulmonary Cd11c+ cells. These results support a likely role for sphingolipid‐mediated pathways in modulating innate immune function during allergic sensitization.

## Methods

### Animals, immunizations, and materials

Seven‐ to eight‐week‐old BALB/c mice were purchased from Jackson Laboratory (Bar Harbor, ME), maintained in a specific pathogen free facility at Cincinnati Children's Hospital Medical Center (CCHMC) and handled under Institutional Animal Care and Use Committee‐approved procedures. Following isoflourane anesthesia, animals underwent allergen immunization as previously described 25 with the following modifications: mice were serially immunized intratracheally every 48 h for 2 weeks with low‐endotoxin, low‐dose HDM extract (*D. pteronyssinus*, Greer Labs; Lenoir, NC) (3 μg total protein/dose in 50 μL of sterile phosphate‐buffered saline plus vehicle [PBS]). Myriocin (Cayman Chemical Company; Ann Arbor, MI) stock solutions (5 mM) were prepared in methanol, and aqueous solutions were prepared fresh before each experiment (1.2 μg/intratracheal dose). Ceramide C6 (N‐hexanoyl‐D‐*erythro*‐sphingosine) was obtained from Avanti Polar Lipids (Alabaster, AL), mouse GM‐CSF was acquired from PeproTech(Rocky Hill, NJ), and RPMI and serine‐free MEM were purchased from Gibco (Carsbad, CA). Normal human bronchial epithelial cells (NHBE) were obtained from Lonza (Walkerville, MD) and cultured per the manufacturer's instructions. Murine bone marrow‐derived dendritic cells (BMDCs) were generated as previously described 26.

### SPT activity assay and steady‐state ceramide quantification

For SPT activity assay, BMDC cells (2 × 10^6^ cells/35 mm plate) were plated and allowed to adhere to polylysine‐coated wells for 12 h in complete RPMI (10% FBS, GM‐CSF 10 ng/mL, 100 U/mL penicillin, 100 μg/mL streptomycin, L‐glutamine 2 mM). Next, cells were incubated overnight in starvation RPMI (0.1% FBS, GM‐CSF 10 ng/mL, 100 U/mL penicillin, 100 μg/mL streptomycin, L‐glutamine 2 mM) and then stimulated with HDM (30ug/mL) for 8 h. Following stimulation, cells were incubated for 2 h in labeling media (serine‐free MEM, 0.1% fatty acid‐free BSA, 5 µCi/mL ^3^H‐serine [PerkinElmer, Waltham, MA]) and then total lipids extracted, ceramides isolated by thin film chromatography (silica gel plates, 60 Å; EMD‐Millipore, Billerica, MA), and SPT activity quantified using a Tri‐Carb 2810TR scintillation counter (PerkinElmer, Waltham, MA), as previously described 12. For lung tissue experiments, mice received intratracheal myriocin (1.2 μg) and were sacrificed 24 h later. Lungs were purged of blood via intraventricular injection of ice‐cold PBS and flash frozen in liquid nitrogen. For NHBE cell ceramide quantification, treated cells were trypsinized, counted, pelleted, and flash frozen. Lipid extraction and ceramide quantification was performed by liquid chromatography‐electronspray ionization‐tandem mass spectroscopy (LC‐ESI‐MS/MS) as previously described 27. For lung and NHBE samples, lipid extract and ceramide quantification was performed by the Lipidomics Core at the Medical University of South Carolina.

### Airway hyper‐responsiveness measurements

Airway resistance was measured in anesthetized, intubated mice with a flexiVent apparatus (SCIREQ; Montreal, Canada) after serial nebulized methacholine challenges, as previously described 25, 28. Briefly, mice were anesthetized with xylazine and sodium pentobarbital and then tracheotomized and cannulated with a blunt, 18‐gauge needle. Mice were ventilated (150 breaths/min, 3.0‐cm water positive end expiratory pressure), and their core body temperatures maintained by a temperature‐regulated heating pad. Methacholine was aerosolized with an ultrasonic nebulizer (Aeroneb; Aerogen, Galway, Ireland), and 20 SnapShot perturbations were performed. The procedure was repeated for 0.01, 6.25, 12.5, and 25 mg/mL concentrations of methacholine. The maximum R‐value with a coefficient of determination of 0.9 or greater (as determined by the flexiVent software) was used to determine the dose–response curve.

### Bronchoalveolar lavage, peripheral blood counts, and cytology

After AHR measurement, blood samples were collected, and bronchoalveolar lavage (BAL) was performed on right lung after clamping left main stem bronchus (Hanks Balanced Salt Solution, Invitrogen; Grand Island, NY). Cells were recovered by centrifugation, BAL fluid (BALF) was frozen, and total cells counted. Slides were stained with Diff‐Quik (Siemens Healthcare; Newark, DE) for differential cell counts. Automated complete blood cell counts (EDTA‐treated peripheral blood) were performed on a Hemavet 950FS (Drew Scientific; Oxford, CT).

### Histology and microscopy

Forty‐eight hours after their final treatment, mice were sacrificed and lung tissue preserved by inflation fixation with 4% paraformaldehyde in PBS. Tissue was then paraffin‐embedded, sectioned (5 µm), and stained (hematoxylin and eosin (H&E) or periodic acid–Schiff stain [PAS]). Specimens were examined and photographed on a B×51 compound microscope and images processed with cellSens software, ver 1.9 (Olympus Corp; Tokyo, Japan). Mean linear density of PAS+ cell was measured in sections from four lung lobes (*n* = 2 mice/group) using Image Pro Plus version 6.1 (Media Cybernetics; Rockville, MD), as previously described 29.

### Lung cell isolation/restimulation

After AHR measurements, the left lung was resected and immune cells isolated as previously described 26. Isolated cells were subsequently cultured in complete RPMI with HDM extract (30 μg/mL) for 72 h.

### Cytokine quantification

BALF CXCL1, CCL11, and IL‐17 concentrations were assessed by a custom multi‐analyte panel (Milliplex) (EMD Millipore; Billerica, MA). Cell culture supernatant cytokines were quantified via enzyme‐linked immunosorbent assay using standard methods and the following kits/reagents: mCCL17 (DY529) and mCXCL1 (DY453) (R&D Systems; Minneapolis, MN); mIL‐5 (Cat Nr: 14‐7052‐81, Cat Nr: 13‐7051‐81, clone TRFK4) and mIL‐13 (Cat Nr: 14‐7133‐81, Cat Nr: 13‐7135‐81) (e‐Biosciences; San Diego, CA); leukotriene B4 (Kit 520111) (Cayman Chem; Ann Arbor, MI); and mC5a antibodies (Clones I52‐1486 and I52‐278) from BD Biosciences (San Jose, CA) and standard (HC1101) from Hycult Biotech (Plymouth Meeting, PA).

### Gene expression

Lung RNA was isolated and cDNA synthesized as previously described 26. Quantitative PCR was performed on a CFX96 Real Time System (BioRad; Hercules, CA). All gene expression studies were performed using TaqMan probes (Applied Biosystems; Carlsbad, CA) and normalized to *Sdha* and *Hprt* via qBase+ software, version3 (Biogazelle; Zwijnaarde, Belgium), with the exception of intelectin 1 and 2, which were performed as previously described 30.

### Pulmonary cell flow cytometry

For ST2+ lymphocyte quantification, lung cells were enriched for CD11b‐/CD19‐ cells by anti‐CD11b and anti‐CD19 microbeads (Miltenyi Biotec; Cologne, Germany) and then stained for Th2 T cell and type 2 innate lymphoid cells as previously described 31. For Cd11c+ lung cell purity, cells were stained with with anti‐mouse Cd11c‐PE antibody (Clone N418) (eBioscience, San Diego, CA). Cells were analyzed on a FACSCanton II flow cytometer (BD Bioscience) and data processed using FlowJo software, version 10 (FlowJo, LLC; Ashland, OR).

### Mouse tracheal epithelial cell (mTEC) culture

Mouse tracheal cells were isolated and cultured as previously described 32. Cultures were supplemented with HDM (30 μg/mL), myriocin (20 μM), or both, and supernatants harvested at 12 and 24 h.

### Pulmonary myeloid cell isolation

Myeloid cells (CD11c+) were isolated from total lung cells via positive selection (murine CD11c kit, StemCell Tech; Vancouver, Canada). Cells were plated (100,000/well) in 96‐well plates with complete RPMI and stimulated with HDM (30 μg/mL), ±myriocin (20 μM). Where noted, cells were pre‐treated with C6 (2 μM) or DMSO (vehicle) for 2 h.

### Statistical analysis

Data are expressed as mean ± SEM. All statistical analyses (unpaired Student's *t*‐test, *T* test with Welch's correction [RT‐PCR data], two‐tailed ANOVA, where appropriate) were performed using Prism, version 6 software (Graphpad; La Jolla, CA).

## Results

### Modulation of *de novo* ceramide synthesis

Condensation of serine and palmitoyl CoA by SPT is the rate‐limiting step in *de novo* ceramide synthesis, and this reaction is inhibited by the potent SPT inhibitor myriocin (Fig. 1A). Macrophages and dendritic cells are known to dynamically regulate intracellular sphingolipid metabolism during activation, increasing their intracellular ceramide concentrations via both sphingomyelin hydrolysis or *de novo* synthesis 18, 19, 33, 34. Using murine BMDCs and radiolabeled serine, we investigated the effects of myriocin (20 μM, 8 h) and allergen exposure (HDM, 30 μg/mL, 8 h) on the *de novo* ceramide pathway (Fig. 1B). Our results confirmed that myriocin strongly inhibits SPT activity, whereas HDM exposure increased SPT catalytic activity. Next, we investigated the effect of myriocin on ceramide steady‐state levels in pulmonary tissues. As expected, *in vitro* myriocin treatment (20 μM, 24 h) significantly reduced total cellular ceramide levels in cultured normal human bronchial epithelial (NHBE) cells (Fig. 1C). To evaluate the *in vivo* effect of myriocin on pulmonary tissue, we quantified ceramide content from whole lung tissue 24 h after a single intratracheal dose of the inhibitor and observed an ∼15% reduction of ceramide per milligram of total protein (Fig. 1D). Further analysis of lung ceramide species by chain‐length showed uniform reduction of most major species, consistent with inhibition of the *de novo* sphingolipid synthesis pathway (Supplemental Fig. S1A). These results show that SPT activity is increased by HDM exposure and that *de novo* ceramide synthesis can be effectively inhibited by myriocin both *in vitro* and *in vivo*.

### Pulmonary SPT inhibition enhances airway hyper‐responsiveness

**Figure 2:**
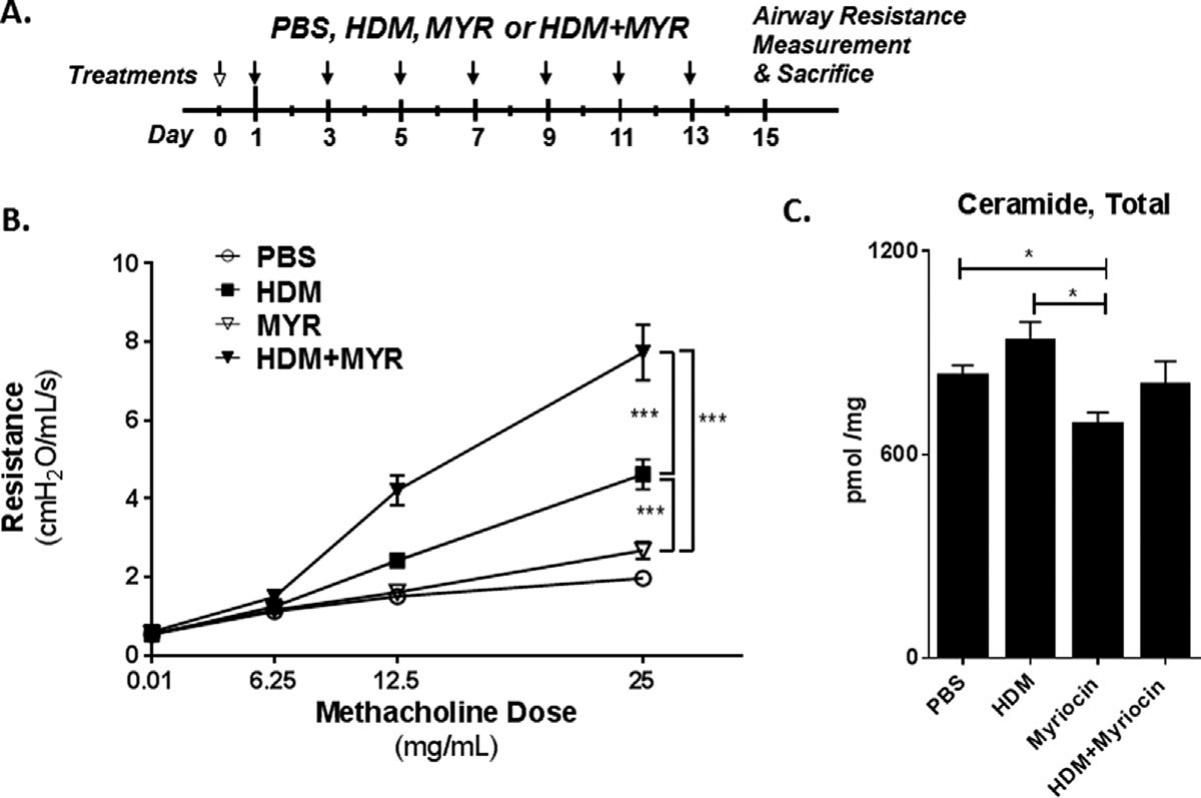
HDM co‐administered with myriocin enhances AHR. (A) Treatment protocol: One day prior to initiation of HDM sensitization, mice received one pre‐treatment dose of either intratracheal vehicle (PBS and HDM groups) or intratracheal myriocin (MYR, HDM + MYR groups; white arrow head day 0). Mice then received seven doses of either intratracheal vehicle (PBS), HDM, MYR, or HDM + MYR every 48 h × 14 days. Mice were sacrificed for AHR measurements 2 days after last treatment. (B) Respiratory system resistance measurements: HDM + MYR significantly increased AHR compared to HDM alone (***p < 0.001, 2‐tailed ANOVA) or MYR alone (***p < 0.001, 2‐tailed ANOVA) (*n* = 20/group, three independent experiments). MYR only treatment did not significantly enhance AHR. (C) Steady‐state total ceramide in whole lung, normalized to total protein, 24 h after final treatment, 2 week protocol. Data are given as mean ± SEM. AHR, airway hyper‐responsiveness; HDM, house dust mite; MYR, myriocin.

**Figure 3:**
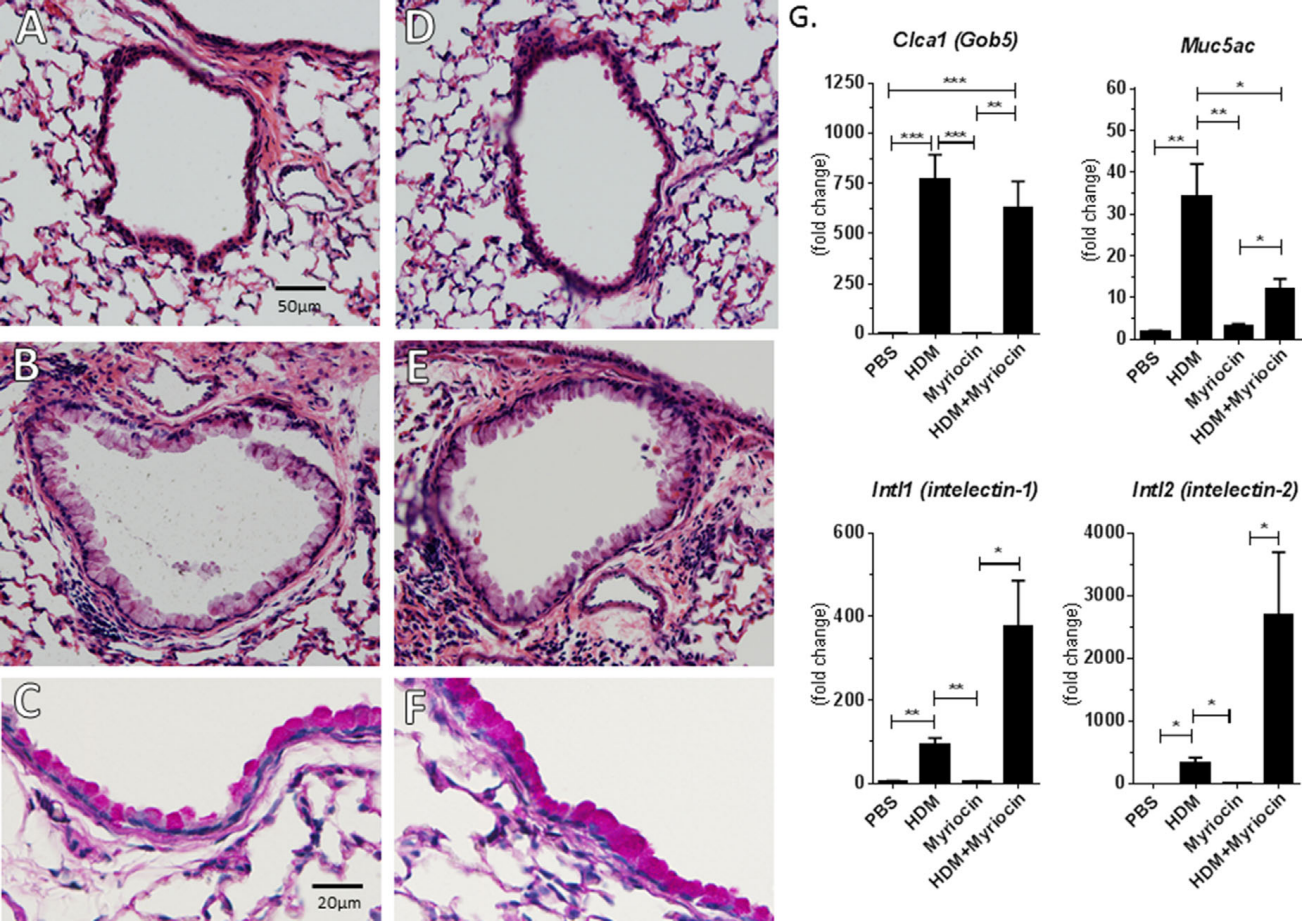
Lung histology and goblet cell gene expression. (A–F) Pulmonary tissue examined after 2‐week protocol revealed little difference between vehicle/PBS‐treated (A) and MYR‐treated (D) mice. HDM‐treated (B and C) and HDM + MYR‐treated (E and F) mice showed similar levels of goblet cell metaplasia and peribronchial infiltrates. (A, B, D, E H&E, 20×). (C and F) PAS staining of HDM‐exposed bronchial epithelium (40×). (G) Whole‐lung gene expression analysis of goblet cell–associated genes. In HDM‐treated mice, there was trend toward increased *Itln1* and *ltln2* expression with MYR co‐treatment, whereas *Clca1/Gob5* was unchanged and *Muc5ac* was reduced by MYR co‐treatment. (*n* = 8 mice/group) (*p < 0.05,**p < 0.01, ***p < 0.001). Data are given as mean ± SEM. HDM, house dust mite; MYR, myriocin.

Allergen‐induced AHR is a robust physiologic end‐point for quantitating the influence of bioactive compounds on the pathophysiology of allergic asthma. Using a well‐characterized murine model of HDM‐induced AHR, we investigated the biochemical and physiologic effects of intratracheal myriocin on lung lipid content, lung function, and airway structure (treatment protocol outlined in Fig. 2A). Similar to other investigators, we administered a low dose of intratracheal HDM extract, which sub‐maximally induces AHR and airway inflammation, thus increasing our ability to detect additive and synergistic effects 25, 35, 36, 37. First, we performed AHR measurement on mice after eight serial doses of intratracheal myriocin and did not detect a significant rise in AHR compared with vehicle‐treated control mice (Fig. 2B) despite a sustained reduction in lung ceramide levels (Fig. 2C). Next, we performed histologic examination of lung tissue from myriocin‐treated mice, which showed normal bronchial and alveolar architecture, similar to vehicle‐treated mice (Fig. 3A vs. D).

After establishing that intratracheal myriocin does not induce significant AHR in our system, we compared the effect of HDM/myriocin co‐administration with that of HDM alone and found that co‐administration significantly enhanced AHR (*p* < 0.001) (Fig. 2B). Myriocin's effect on lung ceramide levels, however, was undetectable in animals co‐treated with HDM/myriocin (Fig. 2C,Supplemental Fig. S1B). Lung histology from both HDM only and HDM/myriocin‐treated mice revealed similar levels of peribronchial inflammatory infiltrates (Fig. 3B vs. E) and goblet cell metaplasia (Fig. 3C vs. F, Supplementary Fig. S1C). Goblet cell gene expression analysis revealed that HDM exposure induced multiple asthma‐associated transcripts, including chloride channel calcium‐activated 1 *(Clca1*/*Gob‐5*), mucin 5, subtypes A and C, tracheobronchial/gastric (*Muc5ac*), intelectin 1 (*Itln1*), and intelectin 2 (*Itln2/B*). Compared to HDM alone, HDM/myriocin co‐administration enhanced *Itln1* and *Itln2* expression, had no effect on *Clca1*, and significantly decreased *Muc5ac* expression. Therefore, in an allergic model of asthma, myriocin‐treatment is associated with enhanced HDM‐induced AHR and an altered goblet cell gene expression pattern.

### Intratracheal myriocin alters airway inflammation

**Figure 4:**
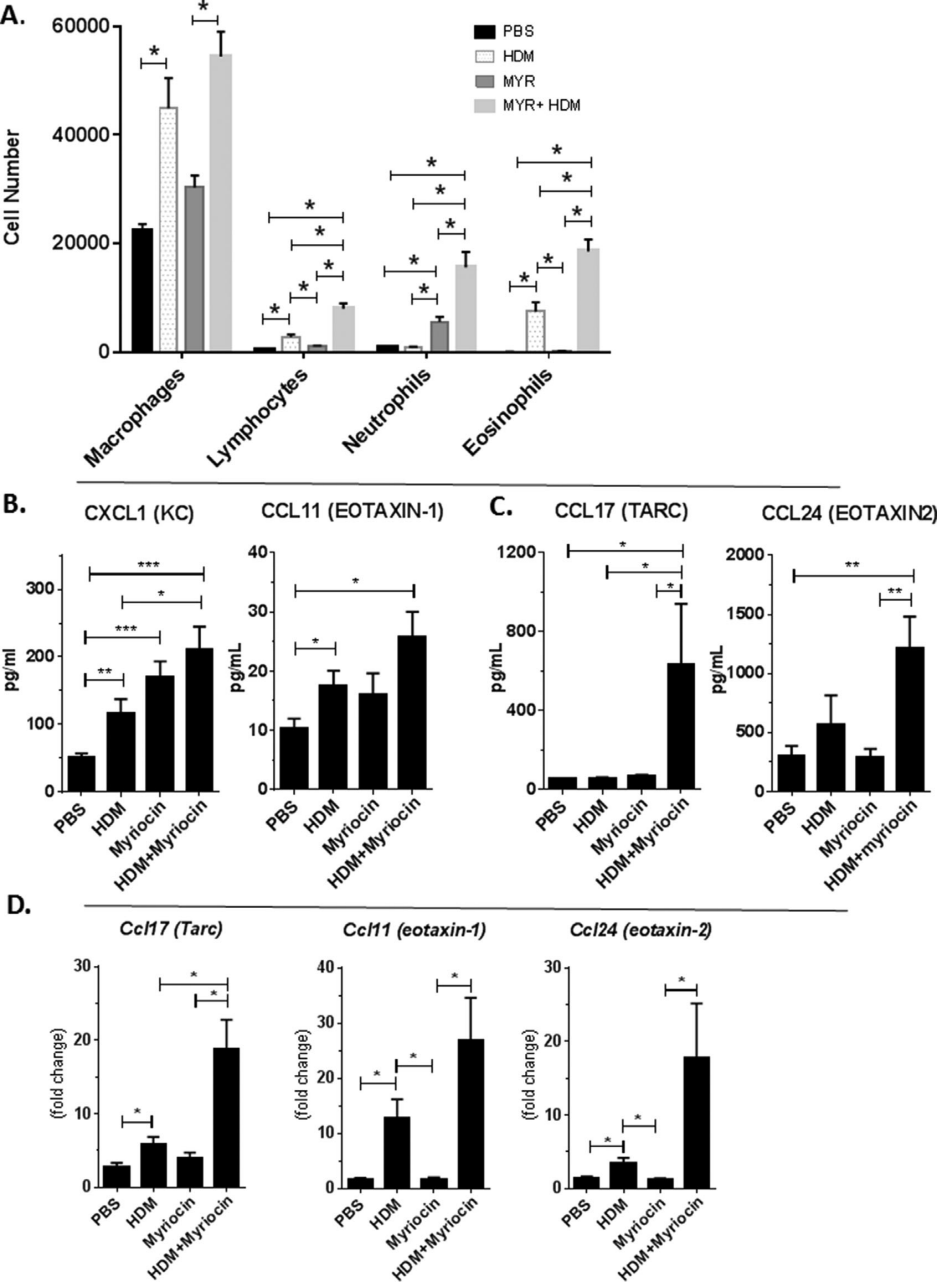
Intratracheal myriocin alters airway inflammatory cell recruitment. (A) BALF cytology analysis shows MYR only significantly increases airway neutrophil counts compared to vehicle, while HDM + MYR increased airway lymphocyte, neutrophils, and eosinophil counts compared to HDM alone. (B) BALF cytokine quantification revealed increased airway CXCL1 concentrations in MYR‐alone and HMD + MYR mice, compared with controls (PBS, HDM‐alone). HDM‐associated increases in CCL11 were also detected (*n* = 14 mice/group). (C) CCL17 and CCL24 supernatant protein levels were increased in cultured lung cells isolated from HDM + MYR treated mice. (D) Chemokine gene expression in whole lung revealed HDM‐associated increases in Ccl17, Ccl11, and Ccl24. HDM + MYR co‐treatment significantly enhanced Ccl17 compared to HDM alone (*n* = 8 mice/group). (*p‐value < 0.05,**p‐value < 0.01, ***p < 0.001, *t*‐test). BALF, bronchial alveolar lavage fluid; IT, intratracheal; HDM, house dust mite; MYR, myriocin.

To further investigate the effects of intratracheal myriocin, we performed BAL on treated mice and analyzed the cellular and chemokine profiles of the recovered fluid. As expected, HDM sensitization increased airway recruitment of macrophages, lymphocytes, and eosinophils (Fig. 4A). HDM/myriocin co‐administration significantly enhanced lymphocyte, neutrophil, and eosinophil airway recruitment compared to HDM alone. Since systemic myriocin administration is reported to induce lymphopenia 38, 39, we performed peripheral blood counts on each treatment group and found no significant differences in peripheral lymphocyte counts (Supplemental Fig. S1B). Of note, myriocin‐only treatment significantly increased airway neutrophil recruitment compared to vehicle‐treated and HDM‐only treated mice (Fig. 4A). This finding is similar to an increase in airway neutrophils observed by Worgall et al. at 3 h post‐intranasal myriocin treatment, although the increase in that study did not reach statistical significance 23. BALF chemokine quantification revealed increased airway concentration of the neutrophil chemotactic factor CXCL1 (a.k.a. keratinocyte chemoattractant [KC] or GROα) in myriocin only‐treated mice compared with vehicle‐treated mice and in HDM/MYR co‐treated mice versus HDM‐only mice (Fig. 4B). We also evaluated the BALF concentrations of additional neutrophil chemotactic factors (leukotriene B4, IL‐17, complement C5a), but none of these varied across our treatment groups (Supplemental Fig. S2A). We also noted that the eosinophil chemokine eotaxin‐1 (CCL11) was increased in BALF from both groups of HDM‐treated mice (Fig. 4B). Supernates from restimulated lungs cells revealed enhanced production of the Th2 T cell chemokine CCL17/TARC (thymus and activation regulated chemokine) and the granulocyte chemokine CCL24/EOTAXIN‐2 in lung cells derived from co‐treated mice (Fig. 4C), thus correspond with the elevated airway lymphocytes and eosinophils observed via BALF cytology.

Finally, we evaluated chemokine gene expression in whole lung samples and found significantly increased expression of the Th2 T cell chemokine *Ccl17 (Tarc*) in HDM + MYR co‐treated mice compared to HDM‐only (Fig. 4D), consistent with the increased airway lymphocyte counts observed in HDM + MYR mice. There was also a trend toward increased expression of the myeloid cell‐derived eosinophil chemokine *eotaxin‐2* (*Ccl24*) in HDM + MYR co‐treated mice compared to HDM‐alone, but this was not statistically significant. Similarly, *eotaxin‐1* (*Ccl11*) expression was also equally enhanced in both HDM‐treated groups. In summary, HDM‐MYR co‐treatment altered airway inflammatory cell recruitment, generating a mixed granulocyte airway infiltrate enriched with neutrophils compared to the eosinophil dominated infiltrates seen with HDM‐only treatment. These myriocin‐associated changes correspond with enhanced airway CXCL1 levels and increased Th2 T cell and myeloid‐cell derived chemokine expression.

### Enhanced Th2 cell recruitment and cytokine production

**Figure 5:**
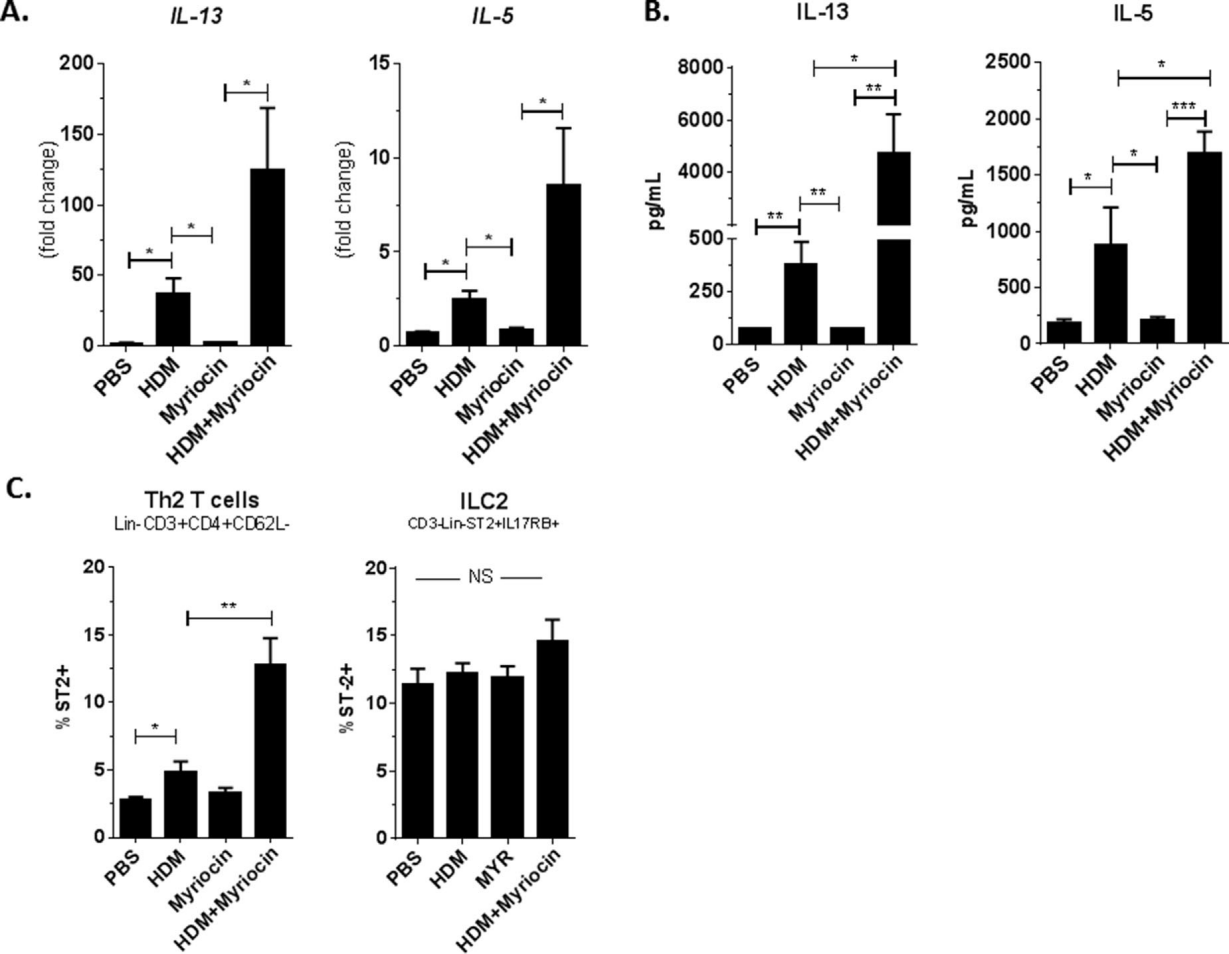
HDM plus MYR co‐treatment enhances Th2 chemotaxis. (A) Th2 effector cytokine gene expression, whole lung. Both *IL‐13* and *IL‐5* were significantly induced following intratracheal HDM treatment compared to vehicle/PBS or MYR alone. (B) IL‐13 and IL‐5 supernate concentrations were significantly increased in lung cells isolated from HDM + MYR treated mice compared to HDM alone. (C) ST2^+^ lymphocyte counts: The frequency of Th2+ T cells (lin‐CD3 + CD4 + CD62L−) were increased in HDM + MYR co‐treated mice compared to HDM alone (**p‐value = 0.003) In contrast, ILC‐2 cell frequency was not significantly altered (*n* = 7/group). Data are given as mean ± SEM. HDM, house dust mite; MYR, myriocin; ILC‐2, innate lymphoid cells, type‐2.

IL‐13 plays a multi‐faceted role in asthma pathogenesis, triggering goblet cell differentiation and altering smooth muscle contractility 40. Similarly, IL‐5 is a critical eosinophil survival associated with allergic asthma. After observing enhanced expression of multiple IL‐13 target genes and noting a significant increase in the numbers of airway lymphocytes and eosinophils in HDM‐myriocin co‐treated mice, we hypothesized that intratracheal myriocin may alter lymphocyte trafficking during allergic sensitization. To assess Th2 cytokine production in our model, we evaluated *IL‐13* and *IL‐5* gene expression in whole lung and quantified Th2 cytokine production in HDM‐restimulated lung cells. *IL‐13* and *IL‐5* transcripts were increased in HDM and HDM‐myriocin treated lungs (Fig. 5A). Supernates from restimulated lungs cells revealed significantly increased IL‐13 and IL‐5 production in cells isolated from HDM + myriocin treated mice compared to HDM‐only mice (Fig. 5B). Myriocin alone did not induce detectable IL‐5 or IL‐13 production. To investigate the source of the increased Th2 cytokines seen in co‐treated mice, we performed pulmonary lymphocytes flow cytometry on post‐treatment lungs and detected a significant increase in the percentage and absolute numbers of Th2 T cells (CD3+/CD4+/CD62L−/ST2+) from HDM‐myriocin co‐treated mice (Fig. 5C, Supplemental Fig. S2B). Group‐2 innate lymphoid cells (ILC2s) are a prominent additional source of Th2 cytokines, but we did not detect a significant increase in this cell population in any of our treatment groups (Fig. 5C, Supplemental Fig. S2B). Together these findings indicate that intratracheal myriocin alters airway allergic inflammation by increasing Th2 T cell recruitment and boosting eosinophil survival and trafficking.

### Airway epithelial chemokine production

**Figure 6:**
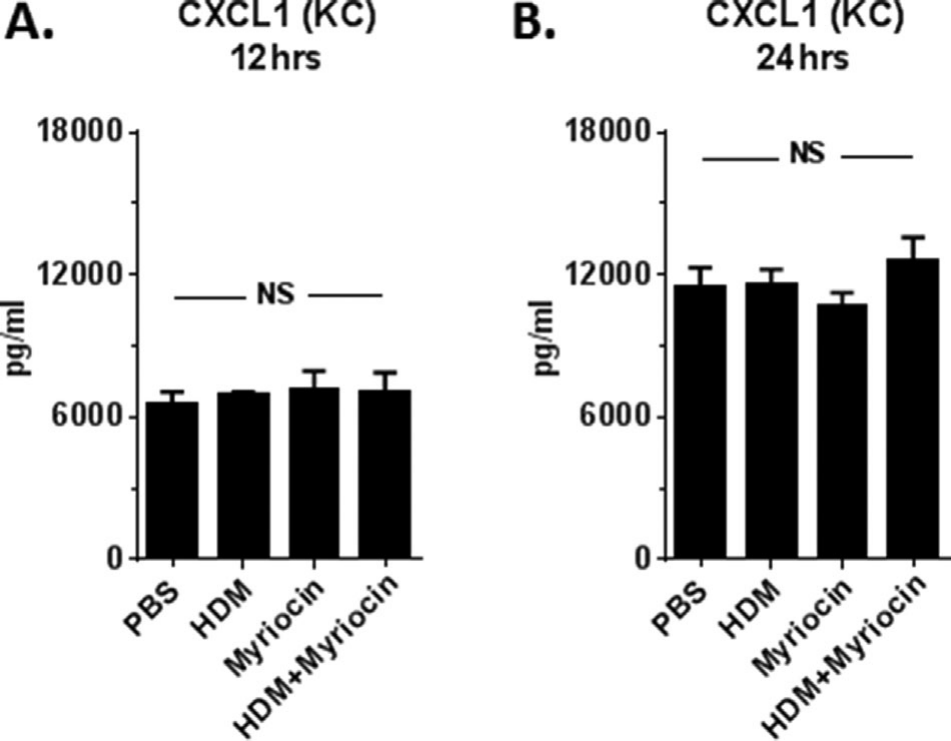
Epithelial cells do not increase CXCL1 production with myriocin co‐treatment. Mouse tracheal epithelial cells (mTEC) were grown to confluence and then stimulated with HDM ± MYR (20 µM) for 12 h (A) or 24 h (B) (performed in triplicate, two independent experiments). No significant enhancement of CXCL1 was noted. Data are given as mean ± SEM. MYR, myriocin.

Both airway epithelial cells and myeloid cells generate chemokines in response to allergen exposure. When combined with HDM airway exposure, intratracheal myriocin amplifies Th2 T cell (CCL17) and granulocyte chemokine (CXCL1, CCL11, CCL24) production. While pulmonary CCL17 and eotaxin‐2 are primarily generated by myeloid cells (dendritic cells, alveolar macrophages), the neutrophil chemokine CXCL1 is more broadly expressed by non‐immune cells, such as the airway epithelium. To assess the effect of myriocin on antigen‐induced epithelial chemokine production, we utilized *in vitro* mouse tracheal epithelium‐derived cultures (mTEC). Surprisingly, CXCL1 supernatant levels were not significantly altered by allergen or myriocin treatment (Fig. 6A and B).

### Pulmonary myeloid cell CXCL1 production

**Figure 7:**
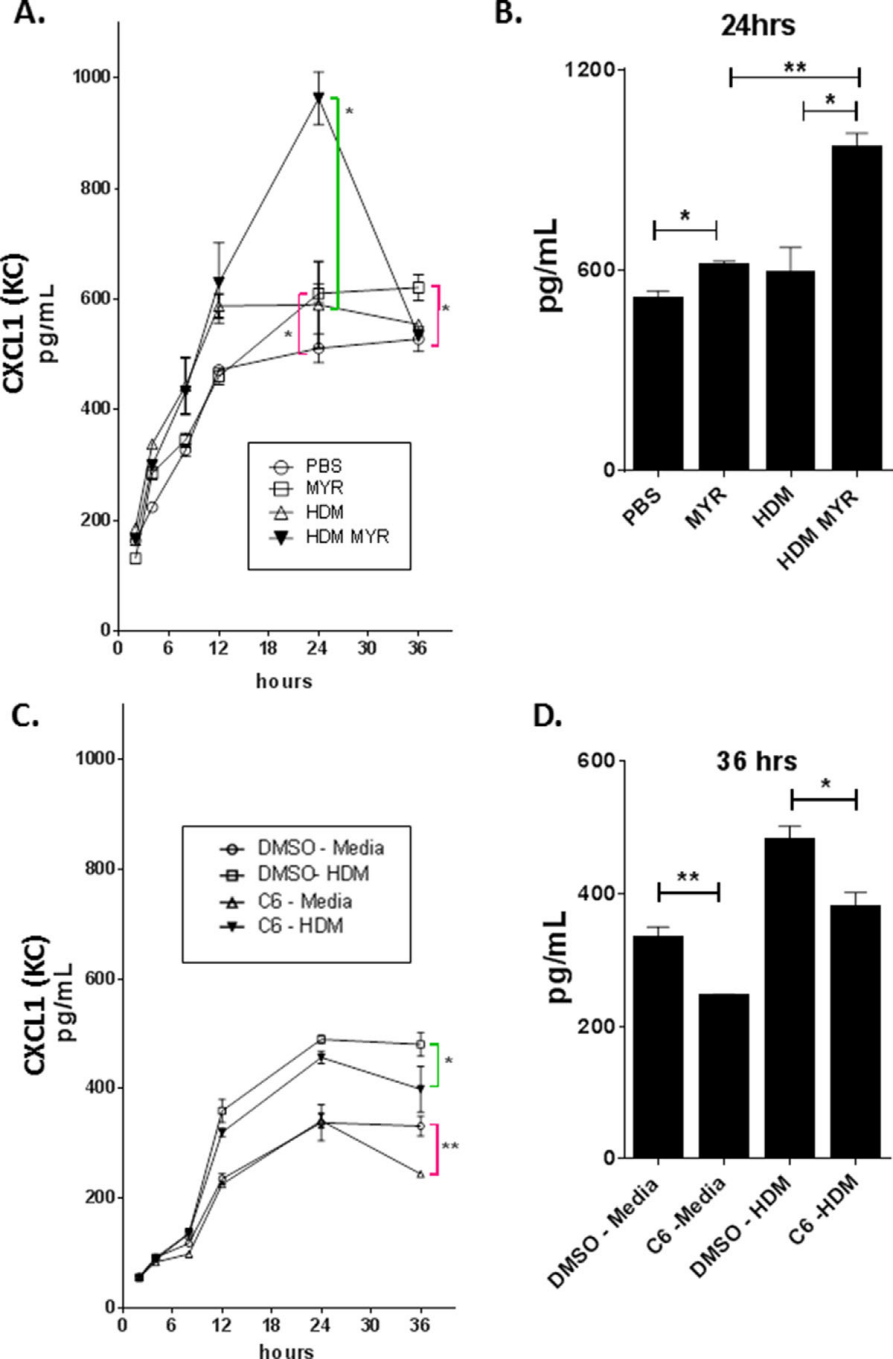
Pulmonary myeloid cell CXCL1 kinetics are regulated by ceramide. (A) CD11c+ cells were stimulated with HDM ± MYR (20 µM) and supernatants collected at indicated time points. MYR alone modestly increased CXCL1 levels at 24 and 36 h compared to vehicle/PBS (*p‐value < 0.05, pink brackets) while HDM + myriocin dramatically increased CXCL1 levels at 24 h (*p‐value = 0.02, green bracket) (*n* = 3/group/timepoint, two independent experiments). (B) Quantification of results from (A) at 24 h. (C) CD11c+ cells were isolated and pre‐treated for 2 h with ceramide C6 or vehicle. Ceramide C6 blunted spontaneous (**p‐value < 0.01, pink bracket) and HDM‐induced CXCL1 production at 36 h (*p‐value <0.02, green bracket). (*n* = 3/group/timepoint, two independent experiments). (D) Quantification of results from (C) at 36 h. HDM, house dust mite.

Pulmonary myeloid cells, especially dendritic cells and alveolar macrophages, are potent sources of inflammatory cytokines 41 and utilize ceramide signaling to modulate their activation states 18, 19. Myriocin‐only intratracheal treatment generated a significant airway neutrophilia via enhanced CXCL1 production (Fig. 4A and B) but airway epithelial CXCL1 production was not significantly enhanced by myriocin treatment. To investigate the effect of myriocin on pulmonary myeloid cells, we isolated CD11c+ pulmonary cells and characterized CXCL1 secretion kinetics following stimulation (Fig. 7, Supplemental Fig. S3B). HDM‐myriocin co‐treatment dramatically enhanced *in vitro* CXCL1 production, while myriocin‐only treatment modestly increase CXCL1 levels (Fig. 7A and B). Flow cytometry of the pulmonary CD11c+ cells used in these experiments confirmed that 95% of these cells were dendritic cells and alveolar macrophages (data not shown). To further clarify the role of ceramide in CXCL1 production, we treated CD11c+ with exogenous ceramide C6 (2 µM) for 2 h prior to HDM‐activation and then analyzed CXCL1 kinetics. We found CXCL1 production was blunted by ceramide pre‐treatment (Fig. 7C and D), supporting an inhibitory role for ceramide signaling in CXCL1 regulation. Myriocin's inhibition of *de novo* ceramide production dysregulates CXCL1 kinetics in myeloid cells.

## Discussion

In the present study, we show that intratracheal myriocin exacerbates airway inflammation and allergen‐induced AHR in a murine model of asthma. Intratracheal myriocin increased immune cell recruitment to the airways by altering chemokine production, especially in the context of allergen sensitization. In addition, enhanced Th2 T cell accumulation in the allergen/myriocin co‐treated lung increased pulmonary cytokine levels (IL‐13 and IL‐5), which exacerbated airway eosinophilia and AHR. While our *in vitro* studies show myriocin and exogenous ceramide treatment have opposing regulatory effects on chemokine production in pulmonary Cd11c^+^ cells, the precise molecular and cellular mechanism(s) by which myriocin enhances pulmonary allergic inflammation *in vivo* remains unresolved. Our whole lung lipid studies show that myriocin‐treated mice had significantly reduced ceramide levels compared to PBS or HDM treated mice. In addition, HDM‐only treated mice showed a trend toward (*p* = 0.07) increased total ceramide levels, driven by a significant increase in the long‐chain ceramide Cer 24:1 (Supp Fig. S1B). Others have also documented similar HDM‐induced increases in whole lung long‐chain ceramides, supporting our finding that HDM exposure alters the pulmonary sphingolipid profile *in vivo* 42. In contrast, in our experiments ceramide levels in HDM + myriocin treated mice were not significantly different from those of PBS mice, suggesting that IT myriocin disrupts HDM‐associated increases in total lung ceramide levels. What remains unclear is if whole organ steady‐state pulmonary ceramide changes are necessary to drive the observed enhancement of the asthma‐like phenotype or whether alterations of sphingolipid metabolism in critical ceramide‐sensitive cellular subpopulations, such as Cd11c+ immune cells, are alone sufficient to drive the amplified pathology. While further investigations beyond the scope of this study will be required to resolve the precise *in vivo* mechanisms driving myriocin‐enhanced allergic disease, increases in pulmonary sphingolipids clearly play a role in a wide variety of lung inflammatory responses (cigarette smoke 22, Pseudomonas aeruginosa pneumonia 43, allergen‐exposure 42). Our findings support the emerging role of ceramide as a counter‐regulatory molecule that modulates inflammatory responses and provide new experimental context to the interpretation of genetic association studies that link ceramide regulatory genes with asthma pathogenesis 6, 9, 21.

Allergic asthma is a complex immunologic and physiologic phenotype resulting from multiple innate and adaptive immune mechanisms 44, 45. We designed our *in vivo* experiments to reduce the inherent complexity of asthma pathogenesis by utilizing intratracheal rather than systemic myriocin dosing, an approach that targets SPT inhibition to pulmonary cells and resident immune cells while minimizing effects on systemic sphingolipid homeostasis. Myriocin's inherently low solubility in aqueous solutions and our intratracheal delivery preferentially expose epithelial and airway‐sampling immune cells (e.g., alveolar macrophages) to high local concentrations of the drug, making these cells the most likely to exhibit direct functional changes that alter airway inflammation. To explore the direct effects of myriocin on these populations, we performed *in vitro* experiments on primary lung epithelial cells (mTEC) and pulmonary antigen‐presenting cells (CD11c^+^ cells). Comparing these cells showed that myriocin treatment significantly increases CXCL1 production in CD11c^+^ cells but not in epithelial cells. These findings demonstrate that CXCL1 production in pulmonary CD11c^+^ cells is dysregulated by SPT inhibition, supporting our hypothesis that these cells directly drive the enhanced airway neutrophilia observed after intratracheal myriocin dosing.

In contrast to airway cells, circulating immune cells (e.g., naïve T cells) and the deeper pulmonary tissues (e.g., smooth muscle cells) receive less myriocin in our *in vivo* system, and thus changes in their function are more likely to be due to indirect or secondary mechanisms. With regard to T cells, a known side effect of systemic myriocin dosing is lymphopenia. In our studies, peripheral blood counts show no change to absolute lymphocyte counts after eight consecutive intratracheal myriocin doses, suggesting that little myriocin was absorbed systemically (Supplemental Fig. S1D). Therefore, the observed increase in pulmonary Th2 cell recruitment after HDM + myriocin co‐treatment can more reasonably be linked to the observed elevation in pulmonary CCL17 production and not to a direct myriocin‐mediated effect on Th2 differentiation, however, such a direct T cell effect cannot be fully ruled out at this time. Intriguingly, genetic deletion of CD11c^+^ cells in a murine model of asthma (recombinant IL‐13‐induced) blocked pulmonary CCL17 production and profoundly reduced airway lymphocyte accumulation 41. It is tempting to speculate that myriocin may directly enhance CCL17 production from CD11c^+^ cells, but we were unable to detect CCL17 production in our *in vitro* cell system without the addition of exogenous GM‐CSF or IL‐4 (data not shown). In contrast, *in vitro* restimulation of total lung cells did generate significant levels of secreted CCL17 without the addition of exogenous factors (Fig. 5B), highlighting the complexity of Th2 chemokine regulation. The role of sphingolipid signaling in CCL17 induction is an intriguing area of ongoing investigation.

Although SPT inhibition clearly has a direct effect on CD11c^+^ cells, myriocin's direct effect on smooth muscle cells is less clear. A study using a very high‐dose intratracheal myriocin treatment (16× the dose used in this report) has reported altered magnesium channel activity in airway smooth muscle cells. These smooth muscle findings were linked to enhanced expression of a specific smooth muscle magnesium channel; however, we did not observe an increase in the expression of the implicated magnesium transport gene *Trpm7* (transient receptor potential cation channel, subfamily M, member 7) in any of our treatment groups (Supplemental Fig. S2C) 23. These differing results may be the result of dosage and timing differences between studies. Our airway resistance measurements were made 48 h after the last myriocin dose (vs. 3 h in the prior study), suggesting that any direct myriocin effect on bronchial constriction may be transient. Nevertheless, our results clearly show that in the context of HDM‐induced disease, elevated Th2 cell counts and smooth muscle hyper‐responsiveness are important consequences of HDM + myriocin co‐treatment. The totality of our evidence, however, supports a model wherein direct dysregulation of ceramide‐mediated innate immune responses initiates the cascade of events that leads to the observed enhancement of airway disease.

Multiple clinical and animal‐based studies support the important role of innate immune responses to allergen exposure. In human studies, pulmonary sub‐segmental bronchial challenges with allergens (house dust mite, pollen) and irritants (diesel particles) have shown that the airways of both asthmatic and non‐asthmatic individuals generate innate inflammatory chemokines/cytokines (IL‐8, TNF‐α, CCL2, CCL5) shortly after exposure; however, the magnitude of these responses are increased in individuals with asthma 46, 47, 48, 49. Interestingly, murine studies have revealed that intratracheal instillation of select chemokines (CXCL1, CCl2, CCL12) is capable of inducing AHR and mucin expression. These chemokines can synergize with IL‐13, leukotrienes, and other pro‐inflammatory mediators to amplify allergen‐triggered airway pathology 45. Although excessive pro‐inflammatory chemokine signaling can exacerbate allergen‐mediated responses, blocking certain chemokine signaling pathways can reduce pulmonary dysfunction. Targeting of CXCL1 or its receptor CXCR2 with blocking antibodies was recently shown to reduce AHR and goblet cell metaplasia in a murine asthma model 50. Therefore, dysregulation of the innate immune responses (e.g., exaggerated chemokine production) may significantly influence the intensity of acute allergen‐induced airway inflammation and hasten the development of asthma‐associated AHR. The enhanced pulmonary CXCL1 production that we observed in myriocin‐treated lungs indicates that regulation of early responses to allergen may have an important upstream effect on asthma pathogenesis.

The direct effects that ceramide signaling plays in the inflammatory responses of myeloid and epithelial cells are complex and multi‐faceted. Our results show that myriocin‐mediated SPT inhibition drives exaggerated chemokine production, especially in myeloid immune cells. These conclusions are consistent with previous observations showing that macrophages and dendritic cells dynamically regulate their sphingolipid metabolisms during activation, increasing their intracellular ceramide concentrations via both sphingomyelin hydrolysis and *de novo* synthesis 18, 19, 33, 34. Multiple studies have shown that such sphingolipid changes have meaningful effects on immune function. For example, increased cellular ceramide drives dendritic cell terminal differentiation, blocking antigen uptake while enhancing peptide presentation 51. In addition, LPS has been shown to increase cellular ceramide and ceramide‐1‐phosphate levels in macrophages, and treatment with exogenous ceramides downregulates TNF‐α and CXCL2 production by these cells 19. Ceramides appear to influence non‐myeloid cell immune responses as well. Neutrophil chemokine IL‐8 (CXCL8) production by a human respiratory epithelial cell line (BEAS‐2B) was found to be regulated by ceramide‐dependent activation of PP2a phosphatase 52. In total, these findings support an immune modulatory role for ceramide production in a variety of immune and non‐immune cells, with the lipid acting as an inhibitory feedback mechanism to attenuate excessive chemokine/cytokine production. Future studies are needed to clarify how dysregulation of endogenous sphingolipid regulatory genes (e.g., *ORMDL3*) may contribute to pathologic airway inflammation.

Like immune cells, airway mucosal cells respond to exogenous allergens and irritants by releasing a diverse array of inflammatory mediators 45. In addition to modulating inflammation, bronchial epithelial cells also influence airway dynamics by signaling to the underlying bronchial smooth muscle and via differentiation into mucin‐secreting goblet cells. IL‐13 signaling plays a central role in goblet cell differentiation and metaplasia by activating STAT6‐ and MAPK‐dependent pathways 53. In our study, mice co‐treated with HDM and myriocin exhibited a trend toward increased expression of the IL‐13‐induced intelectin genes. In contrast, we noted that HDM‐induced *Muc5ac* expression was significantly reduced when myriocin was co‐administered. The mechanism by which myriocin treatment attenuated allergen‐induced *Muc5ac* expression is unclear but may be related to altered sphingolipid signaling in airway epithelial cells. Intriguingly, a recent study showed that production of intracellular sphinosine‐1‐phosphate (S1P), a product of ceramide deacetylation, mediated IL‐13‐driven *Muc5ac* expression in respiratory epithelial cells 54. Additional investigations are currently underway to evaluate the role of sphingolipid metabolism in allergen‐induced goblet cell metaplasia.

In summary, we show that intratracheal myriocin treatment increased airway neutrophil accumulation via enhanced CXCL1 production without affecting AHR. When myriocin was administered during HDM sensitization, the co‐treatment amplified the allergic asthma phenotype (enhancing AHR, Th2 T cell recruitment, and airway eosinophilia) despite failing to reduce steady‐state whole lung ceramide levels. *In vitro* experiments showed that CXCL1 production in antigen‐stimulated pulmonary myeloid cells is regulated by *de novo* ceramide production, suggesting that altered sphingolipid metabolism in these innate immune cells might contribute to asthma pathogenesis independent of changes in global lung ceramide levels. These novel findings indicate that ceramide signaling within key immune cells is an important regulator of allergen‐induced airway inflammation, with implications for the development of new therapies targeting sphingolipid metabolism.

## Conflict of Interest

None declared.

## Supporting information

Additional supporting information may be found in the online version of this article at the publisher's web‐site.

**Figure S1**. Pulmonary ceramide quantification by acyl‐group chain length, 24 h following a single treatment.

**Figure S2**. BALF neutrophil chemotactic Factors: BAL was performed on mice following the completion of 2 week protocol.

**Figure S3**. ST2+ lymphocyte gating strategy.
