# Supplementary figures and images for: Intratracheal myriocin enhances allergen‐induced Th2 inflammation and airway hyper‐responsiveness
Source: Immun Inflamm Dis. 2016 Jun 2;4(3):248–62. doi: 10.1002/iid3.110 (PMC4893390; doi:10.1002/iid3.110)

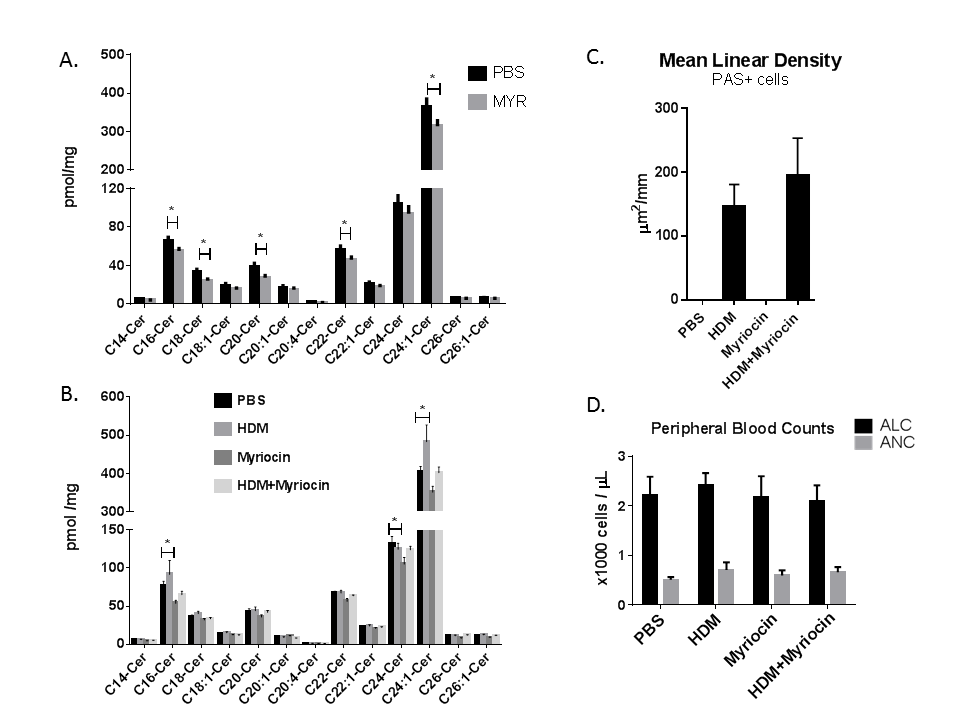

Supplement: Supplementary file 1 — Figure S1. Pulmonary ceramide quantification by acyl‐group chain length, 24 h following a single treatment. [file IID3-4-248-s001.tif]

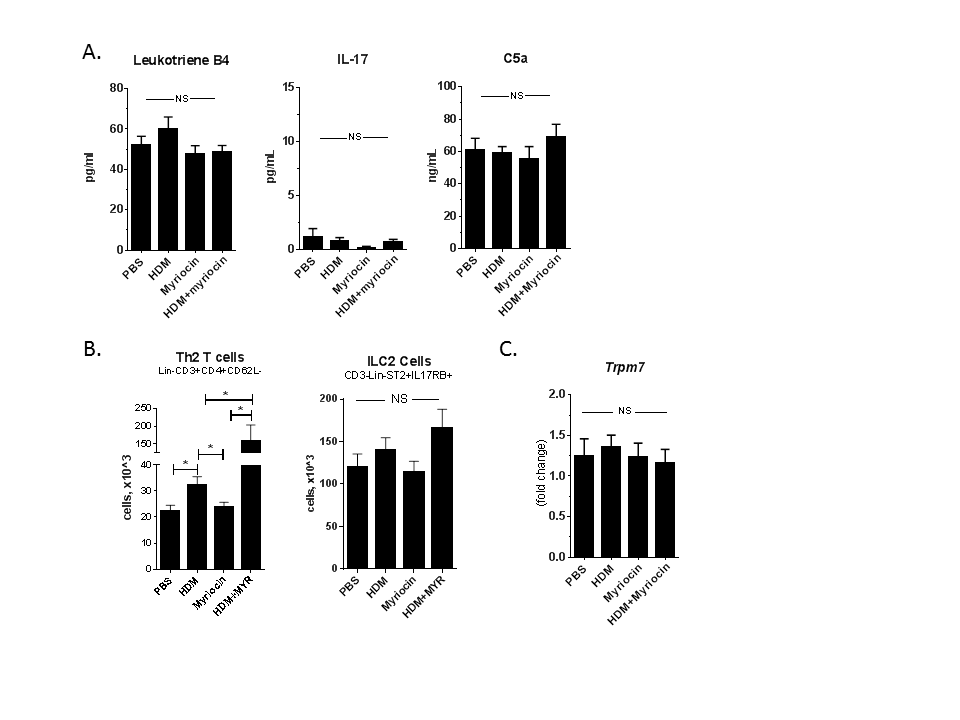

Supplement: Supplementary file 2 — Figure S2. BALF neutrophil chemotactic Factors: BAL was performed on mice following the completion of 2 week protocol. [file IID3-4-248-s002.tif]

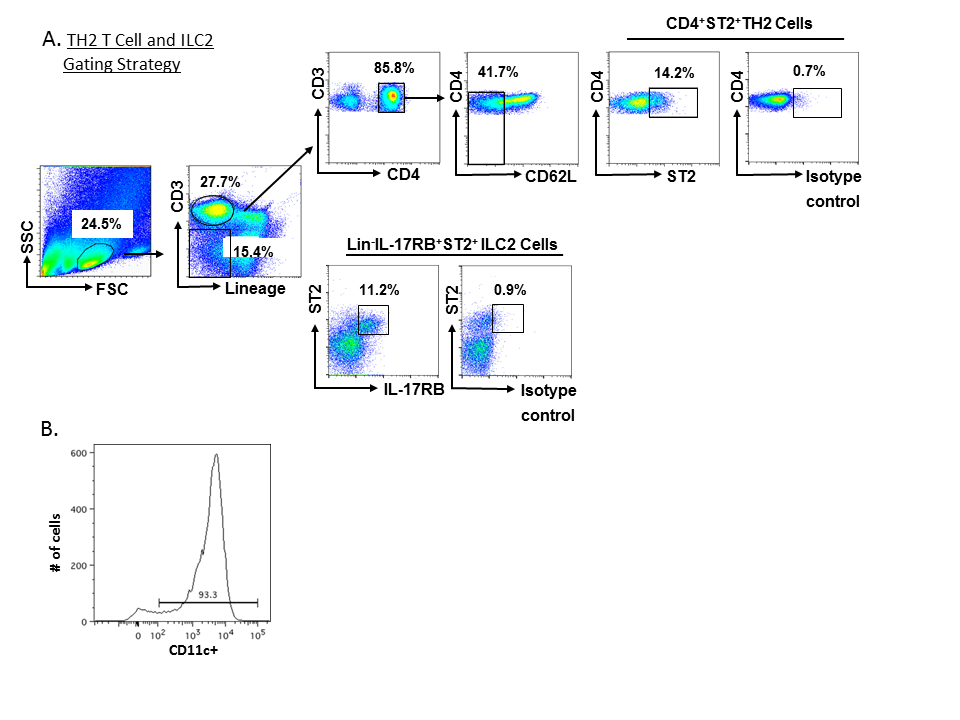

Supplement: Supplementary file 3 — Figure S3. ST2+ lymphocyte gating strategy. [file IID3-4-248-s003.tif]
